# Bimonthly Evolution of Cortical Atrophy in Early Relapsing-Remitting Multiple Sclerosis over 2 Years: A Longitudinal Study

**DOI:** 10.1155/2013/231345

**Published:** 2013-01-10

**Authors:** Robert Zivadinov, Carmen Tekwe, Niels Bergsland, Ondrej Dolezal, Eva Havrdova, Jan Krasensky, Michael G. Dwyer, Zdeněk Seidl, Deepa P. Ramasamy, Manuela Vaneckova, Dana Horakova

**Affiliations:** ^1^Department of Neurology, Buffalo Neuroimaging Analysis Center, The Jacobs Neurological Institute, Buffalo, NY 14203, USA; ^2^Department of Biostatistics, Buffalo Neuroimaging Analysis Center, Buffalo, NY 14203, USA; ^3^Department of Neurology and Center of Clinical Neuroscience, First Faculty of Medicine and General Teaching Hospital, Charles University in Prague, 121 08 Prague, Czech Republic; ^4^Department of Radiology, First Faculty of Medicine and General Teaching Hospital, Charles University in Prague, 121 08 Prague, Czech Republic

## Abstract

We investigated the evolution of cortical atrophy in patients with early relapsing-remitting (RR) multiple sclerosis (MS) and its association with lesion volume (LV) accumulation and disability progression. 136 of 181 RRMS patients who participated in the Avonex-Steroids-Azathioprine study were assessed bimonthly for clinical and MRI outcomes over 2 years. MS patients with disease duration (DD) at baseline of ≤24 months were classified in the early group (DD of 1.2 years, *n* = 37), while patients with DD > 24 months were classified in the late group (DD of 7.1 years, *n* = 99). Mixed effect model analysis was used to investigate the associations. Significant changes in whole brain volume (WBV) (*P* < 0.001), cortical volume (CV) (*P* < 0.001), and in T2-LV (*P* < 0.001) were detected. No significant MRI percent change differences were detected between early and late DD groups over 2 years, except for increased T2-LV accumulation between baseline and year 2 in the early DD group (*P* < 0.01). No significant associations were found between changes in T2-LV and CV over the followup. Change in CV was related to the disability progression over the 2 years, after adjusting for DD (*P* = 0.01). Significant cortical atrophy, independent of T2-LV accumulation, occurs in early RRMS over 2 years, and it is associated with the disability progression.

## 1. Introduction

Multiple sclerosis (MS) is an autoimmune disease of the central nervous system (CNS) that affects both white matter (WM) and gray matter (GM).

In the last decade, there has been increased interest in studying GM damage in MS, especially in the cortical regions [1]. Advances in both MRI acquisition and analysis techniques have enabled better detection of changes in GM morphology [2]. The MRI assessments included measurements of cortical atrophy, cortical thinning, and cortical lesions [1–4].

Because imaging techniques are still unable to adequately detect GM lesions, especially in the cortex [5–9], measurement of cortical atrophy is gaining increasing attention in the literature [1], in order to assess the real extent of cortical pathology in vivo in patients with MS [2].

Recent studies have established that subcortical, but not cortical, atrophy is present at the earliest clinical stages of the disease [10–15]. However, most of these studies had a cross-sectional design, and only a few longitudinal studies investigated possible associations between GM atrophy and clinical outcomes in patients with MS [11, 15–18]. In addition, only one serial MRI study investigated the evolution of GM atrophy over a 9-month period [19]. Therefore, one of the main goals of this study was to investigate the bimonthly evolution of cortical atrophy in patients with early relapsing-remitting (RR) MS over a period of 2 years.

Inflammatory activity in the WM is histopathologically different from changes in the GM [4, 20, 21]. Some of the processes in the cortex even precede changes in WM and are probably more responsible for development of irreversible clinical disability [15–19, 22–24]. Very few longitudinal studies explored the relationship between cortical atrophy development and WM lesion burden accumulation and disability progression in early RRMS [11, 18]. Therefore, another aim of this study was to investigate the effect of cortical atrophy development on disability progression, using bimonthly serial MRI assessments. We also assessed the relationship between accumulation of WM lesion burden and development of cortical atrophy.

## 2. Methods

### 2.1. Patients

MRI data were obtained from patients who were initially enrolled into the 2-year, double-blind, placebo-controlled Avonex-Steroids-Azathioprine (ASA) study [25]. In this study, 181 patients were randomized to treatment with intramuscular (IM) interferon beta (IFN*β*) 1a (30 *μ*g/week) alone or in combination with azathioprine (50 mg/day) or azathioprine plus prednisone (10 mg every other day). Full details of the design, inclusion and exclusion criteria and results of the 2-year study (by randomized treatment group) [25], and preliminary results from the extension 5-year period (by disability progression) were previously reported [18, 26]. In the original study there were no significant differences regarding clinical or MRI outcomes during the 2 years, except the change from baseline in T2-LV that favored the triple-agent combination therapy versus IFN*β*-1a monotherapy (*P* = 0.015) [25]. However, a recent 6-year followup evaluation of the ASA study by original treatment arm reported no significant difference in the absolute T2-LV and its changes over 6 years between the original treatment groups [27], suggesting that original percent change of T2-LV differences were probably inflated by the lower baseline T2-LV in the IM IFN*β*-1a alone group. Therefore, the present study represents a post-hoc analysis of combined patients who had complete clinical and MRI assessments and who participated in a serial MRI substudy over 2 years.

Both MRI and standard clinical assessments were conducted at baseline at 2, 4, 6, 8, 10, 12, 14, 16, 18, 20, 22, and 24 months (13 time-points) over a two year period. The sustained disability progression was assessed at 27 months and was defined as a ≥1.0-point sustained (12-week) increase in the EDSS score in patients who had a baseline EDSS score of ≥1.0 or a ≥1.5-point sustained (12-week) increase in the EDSS score in patients who had a baseline EDSS score of 0.0.

The study was approved by the Medical Ethics Committee of the General University Hospital and the First Faculty of Medicine, Charles University in Prague, Czech Republic, and by the University at Buffalo, NY, USA.

### 2.2. MRI Imaging and Analysis

All MRI assessments were performed using the same Philips Gyroscan 1.5-Tesla scanner (Philips Medical Systems, Best, The Netherlands), located in the Department of Radiology at Charles University in Prague. Axial images of the brain were acquired using fast fluid-attenuated inversion recovery (FLAIR) with 1.5-mm slice thickness and axial T1-weighted 3-dimensional (3D) spoiled gradient-recalled (SPGR) images with 1 mm slice thickness. All images were nongapped. MRI assessment protocols were conducted as previously reported [18, 25, 26].

All serial bimonthly MRI scans (2, 4, 6, 8, 10, 12, 14, 16, 18, 20, 22, and 24 months) were analyzed by the Buffalo Neuroimaging Analysis Center at the Department of Neurology, University at Buffalo, NY, USA. Investigators performing the image analyses were blinded to subject characteristics and clinical or treatment status.

T2 lesion volumes (LVs) were calculated using a reliable contouring-thresholding technique, as previously reported [28]. Volumetric measures were determined on T1-weighted 3D SPGR images that were modified by using an in-house developed inpainting technique to avoid tissue misclassification [28]. Structural image evaluation using normalization of atrophy cross-sectional (SIENAX) [29] was used to obtain normalized whole brain volume (WBV) and cortical volume (CV). For longitudinal changes of the WBV, we applied the SIENA method [29].

### 2.3. Statistical Analysis

Statistical analysis was performed using SPSS 13.0 (SPSS, Chicago, IL, USA) and SAS 9.0 (SAS Institute, Raleigh, NC, USA).

MS patients were classified into two groups based on their disease duration at baseline. Patients with disease duration of ≤24 months were considered the early group, while patients with disease duration lasting >24 months were considered the late group. Thirty-seven patients were classified into the early group, while 99 patients were classified into the late group. The demographic, clinical, and MRI characteristics at baseline and over the followup were explored for the total MS cohort, as well as the early and late disease duration groups. The statistical differences at baseline and at follow-up between the early and late disease duration groups were calculated using the chi-square test, Student's *t*-test, and Mann-Whitney rank-sum test, as appropriate.

The Wilcoxon signed-rank test was used to test for any significant changes within individual groups between the baseline and year 1 and year 2 measurements. Bimonthly within individual group changes in CV were calculated using the Kruskal-Wallis test.

Due to the repeated measures and the nature of the serial MRI data acquisition time points (bimonthly), and in order to estimate the longitudinal effect of cortical atrophy in relation to lesion burden accumulation and disability progression, we used regression (for baseline) and mixed effect model (for followup) analyses [30]. The analysis was performed separately for the total MS cohort and early and late disease duration groups.

In order to avoid too many spurious findings due to multiple comparisons, we do not report anything as statistically significant unless the nominal *P* value was ≤0.01 by using two-tailed tests.

## 3. Results

### 3.1. Demographic, Clinical, and MRI Characteristics at Baseline

Table 1 shows demographic, clinical, and MRI characteristics of the total MS cohort (*n* = 136), as well as of the early (*n* = 37) and late (*n* = 99) disease duration groups. No significant differences were found in baseline demographic, clinical and MRI characteristics between the MS cohort who participated in serial bimonthly MRI ASA study (*n* = 136) compared to the total MS cohort participating in the ASA study (*n* = 181).

Mean disease duration at baseline was 5.5 years for all the patients, with 1.2 years among the early and 7.1 years among the late groups (*P* < 0.0001). As expected, the early disease duration group had a significantly lower age at baseline (27.3 years) compared to the late group (32.2 years). No differences were observed between the two groups in age at onset. The mean EDSS at baseline was 1.9 among all patients in the study, with the late disease duration group having a significantly increased EDSS compared to the early one (*P* = 0.002).

The late disease duration group had a significantly decreased normalized CV (*P* = 0.002), normalized WBV (*P* = 0.01), and increased T2-LV (*P* = 0.01) at baseline compared to the early group.

There were no significant differences in the original treatment arm frequency distribution (IFN*β*-1a alone, IFN*β*-1a + AZA, and IFN*β*-1a + AZA + Prednisone) between the early (32%, 33% and 35%, resp.,) and late (32%, 34%, and 34%, resp.,) disease duration groups.

### 3.2. MRI Outcomes Over the Followup

Significant differences within individual total, early, and late disease duration groups were detected for % changes in WBV (*P* < 0.001) and CV (*P* < 0.001) between baseline and year 1 and baseline and year 2 (Table 2). In the total MS cohort, there was no significant increase in T2-LV between baseline and year 1 only, while there was a significant increase within individual total, early, and late disease duration groups between year 1 and year 2 and baseline and year 2 (*P* < 0.001).

The highest % changes in MRI measures were detected in the early disease duration group between baseline and year 2 for CV (−2.48%), WBV (−2.11%), and T2-LV (48.3%). However, only the increase in T2-LV between baseline and year 2 resulted in a significant difference between the early and late disease duration groups (*P* < 0.01).

**Figure 1:**
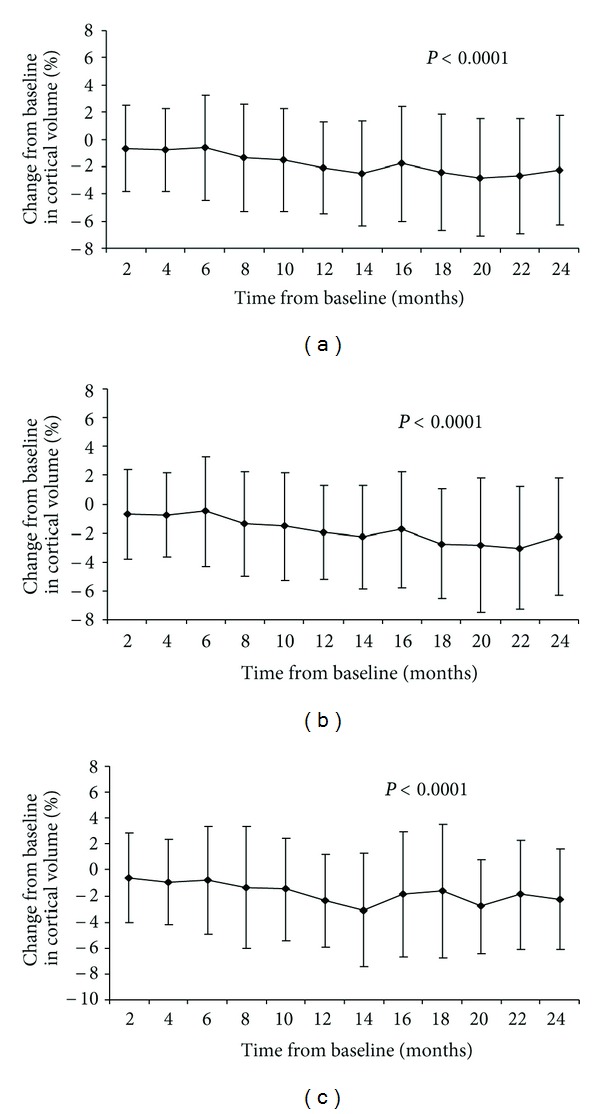
Bimonthly cortical volume percent changes in total multiple sclerosis cohort (a), early (b), and late (c) disease duration groups.

Evolution of bimonthly % CV changes is shown in Figure 1. Significant differences within individual total, early, and late disease duration groups were detected in CV over 2 years (*P* < 0.0001).

### 3.3. Bimonthly Effect of T2-LV Accumulation on Cortical Volume Changes

Table 3 represents the regression (baseline) and mixed effect model (year 1 and year 2) analyses results between T2-LV and CV for the total MS cohort, using all serial MRI time points of the study. After adjusting for patients' age and disease duration, we found that higher T2-LV at baseline was strongly related to decreased CV at baseline (*P* < 0.0001). Nevertheless, the % change in T2-LV at year 1 was not significantly related to the % change in CV over 1 year. Similarly, the change in T2-LV was not significantly related to the change in CV between year 1 and year 2 or baseline to year 2.

Similar analyses were also performed in the two disease duration groups (early and late) both with and without age as a covariate. No significant associations between changes in T2-LV and CV were found in either of these groups over the followup.

### 3.4. Bimonthly Effect of Cortical Volume Changes on Disability Progression

The results from the mixed effect model analysis between CV, WBV, and T2-LV bimonthly changes and disability progression over 2 years are summarized in Table 4. Based on the models, the bimonthly change in CV was significantly related to disability progression over the 2-year study, after adjusting for disease duration (*P* = 0.01). However, a similar model showed that WBV and T2-LV were not significantly related to disability progression over 2 years.

Similar analyses were also performed in the two disease duration groups (early and late) both with and without age, as a covariate. Significant associations between changes in CV and disability progression were found over the followup in both the early and late disease duration groups (*P* = 0.01).

When adjusted for baseline CV, by using the changes in CV from baseline and every six months in our analysis, the association with disability progression became nonsignificant in both early and late RRMS groups.

## 4. Discussion

This study showed that significant bimonthly cortical atrophy occurs in patients with early RRMS over 2 years. This finding is in line with a previous serial MRI study that investigated the evolution of GM atrophy over a 9-month period [19]. The development of cortical atrophy over the 2-year followup was independent of T2-LV accumulation, and was significantly associated with disability progression. No significant MRI changes between early and late disease duration groups were detected over 2 years, except for increased T2-LV accumulation between baseline and year 2 in the early disease duration group.

We performed this study as an exploratory study, and given that we did not know how many comparisons we would make a priori, we did not adjust the significance tests for multiple comparisons. Potential associations identified in the current study may drive future research with preplanned analyses to validate the results of this exploratory research.

The ASA was an investigator-initiated, prospective, longitudinal, double-blind, placebo-controlled study that was originally designed to be 2 years in duration, but due to slow recruitment, it was extended to a 5-year double-blind, placebo-controlled design [18, 25, 26] and to a 10-year open-label phase follow-up [27]. At the 5-year followup, 162 of 181 patients participating in the study had complete clinical and MRI followup. MRI assessments after the first 2 years of the study were performed annually and the clinical examinations quarterly up to year 10. The 10-year followup of all subjects originally enrolled in the study is scheduled to be completed in late 2013. The first 136 of 181 patients enrolled in the ASA study participated in a bimonthly serial MRI study that aimed to investigate early changes of MRI outcomes in relation to short- and long-term clinical outcomes over 2, 5, and 10 years.

One of the main goals in the present study was to examine the changes in cortical atrophy in patients with disease duration of ≤24 or >24 months via the use of bimonthly MRI scanning. An advantage of using the random effects mixed model is that it allows each subject observed in the study to have its own mean, rather than assuming that all subjects in the study have the same mean structure over the course of the study. Our initial hypothesis was that, based on a recently published study, [15] the evolution of cortical atrophy will be ongoing at a slower rate in the early, compared to the late, disease duration group. In that study, it was found that 212 patients with clinically isolated syndrome at first clinical onset had similar CV to the 177 early RRMS participating in the ASA study that were of the similar age, although they had less than 5 years of disease duration. In the present study, the late disease duration group showed that significantly decreased CV compared to the early group at baseline. However, there was almost 5 years of age difference between the early and late disease duration groups. Over the 2-year followup, both groups developed significant cortical atrophy (Figure 1) and the early group showed a slightly increased, though not significant, rate (−2.48%) compared to the late group (−2.12%). These findings suggest that cortical atrophy develops at a similar rate in RRMS patients with early and late disease duration. The current and previous study [15] indicate that age may play an important role in development of cortical atrophy in MS patients and should be taken into account in future studies.

Placebo-controlled clinical studies have shown that IM IFN*β*-1a reduces T2 LV [31], slows disability progression, [32], and prevents development of brain atrophy [33–36]. However, despite these known effects of treatment with IM IFN*β*-1a, both the early and late disease duration groups of MS patients exhibited loss of brain tissue and, in particular, developed cortical atrophy over the 2 years in the present study. However, the present study showed most of the WB and CV volume changes occurred in the first year of the study (Table 2). This phenomenon could be related to a “pseudoatrophy effect,” which was described in the first year of the study in most of recent MS clinical trials using disease-modifying treatment [37]. This could have contributed to accelerated volume changes observed in the WB and CV volumes in the first year in the present study. In the second year of the study, a treatment effect may have taken place, as previously described with use of IM IFN*β*-1a [35]. The extent of cortical atrophy development in untreated MS patients is likely to be greater than that observed in the treated patients in this study. A previous case-control study suggested that IM IFN*β*-1a may slow down GM atrophy progression [36].

GM atrophy development is a better indicator of disability progression than accumulation of lesion burden or WM atrophy [16–18]. It has been shown that cognitive impairment in patients with RRMS is thought to be associated with MS-related GM pathology, particularly in the cortex [38], with variability in disability progression between patients with RRMS possibly arising from differences in GM injury. In the present study, we found, in a mixed effect model analysis that included all bimonthly serial MRI time points over 24 months and corrected for disease duration, that cortical atrophy was significantly associated with development of disability progression, as determined by 12-week sustained worsening at month 27. However, when adjusted for baseline CV, by using the changes in CV from baseline and every six months in our analysis, this association became nonsignificant in both study groups. This finding suggests that cortical atrophy at baseline plays an important role in subsequent disability development over 2 years. As such, the study may have important implication for identifying patients at higher risk for disability progression. On the other hand, development of whole brain atrophy or accumulation of T2-LV over the same period did not show a significant association. The findings were similar in both the early and late disease duration groups. This is indeed an important finding, as it suggests that disability progression over 2 years in the early phase of RRMS may be predominantly related to cortical atrophy.

In the mixed effect model analysis, corrected for age and disease duration, no association was found between development of cortical atrophy and accumulation of lesion burden, when all bimonthly serial MRI time points over 24 months were included. Separate models showed no different outcomes, when only age was used as a covariate, in models that were run on the early or late disease duration groups. This finding indicates that cortical atrophy develops independently of T2-LV accumulation over 2 years. However, in regression analysis, there was a robust relationship between the amount of cortical atrophy and lesion burden at baseline. This indicates that development of cortical atrophy may be associated with T2-LV accumulation, but that a longer period of time may be needed to assess this relationship longitudinally. However, the 5-year results of the preliminary extension of the ASA study [18, 26] argue against an association of T2-LV accumulation with GM atrophy even over a 5-year followup. Although the current study did not use DIR for detection of cortical lesions, the 2D-FLAIR sequence that was used had 1.5 mm thickness. It has been shown previously that this sequence has higher sensitivity for detection of cortical and WM lesions than standard MRI protocol [23].

Although there are several strengths to this study, including bimonthly serial MRI acquisition and prospective and longitudinal design of the study, there are important limits to be considered. The choice of the classification of early RRMS patients in the early and late disease duration groups was arbitrary, as our goal was to investigate whether patients with disease duration of less than 24 months had evolution of cortical atrophy similar to those with more than 24 months. This created two groups of uneven sample size (37 versus 99 subjects) and relapse activity in the previous year that could have influenced to some extent our findings. It could be that other classification criteria, for example, by median disease duration (3.9 years), could have yielded different results. However, this is unlikely, as the MRI changes in individual and total groups were very similar. Use of DIR could also have helped to better estimate the amount of cortical lesion pathology [5–9]; however, when this study was designed, DIR was not yet available. We did not investigate cognitive outcomes in this study. Neuropsychological assessment would be potentially more relevant for association with cortical atrophy, especially in archicortical areas [39, 40].

In conclusion, significant cortical atrophy, independent of T2-LV accumulation, occurs in early RRMS over 2 years, and it is associated with disability progression.

## Figures and Tables

**Table 1:** Descriptive statistics of demographic, clinical, and MRI variables at baseline.

	Total (*n* = 136)	Early group (*n* = 37)	Late group (*n* = 99)	*P* value*
Sex, females (%)	108 (79.4%)	32 (86.5)	76 (76.8)	0.243
Age in years, mean (SD) min–max	30.9 (7.9) 19–56	27.3 (6.7) 19–50	32.2 (7.9) 19.6–56	0.001
Age at onset in years, mean (SD) min–max	23.9 (7.1) 12–51	24.8 (6.5) 18–48	23.5 (7.3) 12–51	0.345
Annual relapse rate, mean (SD) min–max	1.8 (0.8) 0–4	2 (0.83) 0–4	1.8 (0.7) 0–3	0.064
Disease duration in years, mean (SD) median (min–max)	5.5 (5.2) 3.9 ( 0.6–32.5)	1.24 (0.37) 1.2 (0.6–2)	7.1 (5.3) 5.4 (2.1–32.5)	<0.0001
EDSS, mean (SD) median (min–max)	1.9 (0.9) 2.0 (0–4.0)	1.5 (0.9) 1.5 (0–3.5)	2.1 (0.9) 2.0 (0–4.0)	0.002
Normalized WBV, mean (SD) min–max	1494 (76.7) 1303.1–1667.1	1524.3 (64.8) 1393.3–1649.8	1482.6 (77.9) 1303.1–1667.1	0.014
Normalized CV, mean (SD) min–max	587.5 (44.2) 478.9–685.9	608 (34.7) 537.8–685.9	579.8 (45.1) 478.8–670.3	0.002
T2-LV, mean (SD) min–max	10 (11.1) 0.1–58.3	5.9 (6.4) 0.1–24.2	11.5 (12.1) 0.2–58.3	0.01

EDSS: expanded disability status scale, WBV: whole brain volume, CV: cortical volume, and LV: lesion volume.

Annual relapse rate refers to the mean number of relapses in the year before baseline.

The statistical differences between early and late disease duration groups (*P* value*) were calculated using the chi-square test, Student's *t*-test, and Mann-Whitney rank-sum test, as appropriate.

The volumes are reported in milliliters.

**Table 2:** Changes in MRI outcomes over 2 years between different study groups.

	Total (*n* = 136) mean (SD) median	Early group (*n* = 37) mean (SD) median	Late group (*n* = 99) mean (SD) median	*P* value*
% Change in WBV (0-1 years)	−1.59 (3.9) −1.32***	−2.11 (3.5) −1.84***	−1.39 (4.1) −1.1***	0.531
% Change in WBV (1-2 years)	0.12 (5.3) −0.34	0.05 (3.4) −0.35	0.15 (5.9) −0.23	0.428
% Change in WBV (0–2 years)	−1.61 (3) −.37***	−2.23 (2.8) −2.2***	−1.37 (3.1) −0.98***	0.751
% Change in CV (0-1 years)	−2.15 (4.3) 1.78***	−2.31 (3.5) −1.88***	−2.1 (4.6) −1.77***	0.559
% Change in CV (1-2 years)	−0.01 (6.9) −0.09	−0.53 (4.7) −1.36	0.21 (6.6) −0.06	0.140
% Change in CV (0–2 years)	−2.22 (4) −2.28***	−2.48 (4) −2.98***	−2.12 (4) −1.98***	0.606
% Change in T2-LV (0-1 years)	9.3 (43.6) 5.6*	11.8 (48.5) 9	8.8 (40.6) 4.7	0.442
% Change in T2-LV (1-2 years)	28.1 (47.6) 19.5***	38.6 (47.7) 29.5***	24 (47) 16.1***	0.091
% Change in T2-LV (0–2 years)	33.9 (52.8) 24.4***	48.3 (57.5) 41.8***	28.3 (49.9) 19.8***	0.015

WBV: whole brain volume, CV: cortical volume, and LV: lesion volume.

Statistical differences between the early and late disease duration groups (*P* value*) were calculated using Student's *t*-test.

The Wilcoxon signed-rank test was used to test within group % changes of the paired measures involving the baseline and the followup indices (***P < 0.001, **P < 0.01, and *P < 0.05).

**Table 3:** Relationship between cortical volume at baseline and changes with lesion burden over the followup in patients with relapsing-remitting multiple sclerosis.

CV	Coefficient	Standard error	*T*	*P* value
Baseline				
Age at baseline	−2373.48	438.79	−5.41	<0.0001
Disease duration	−608.11	680.62	−0.89	0.37
T2-LV	−1.39	0.28	−4.93	<0.0001
Baseline to year 1				
Age at baseline	−0.005	0.06	0.09	0.93
Disease duration	−0.04	0.08	−0.48	0.63
% Change T2-LV	0.005	0.009	0.52	0.61
Year 1 to year 2				
Age at baseline	0.04	0.05	0.70	0.48
Disease duration	0.08	0.08	1.02	0.31
% Change T2-LV	0.01	0.007	1.51	0.13
Baseline to year 2				
Age at baseline	0.03	0.03	0.90	0.37
Disease duration	0.008	0.05	0.15	0.88
% Change T2-LV	0.002	0.004	0.74	0.46

LV: lesion volume; CV: cortical volume.

The statistical analysis between T2-LV and CV was performed using regression (baseline) and mixed effect model (year 1 and year 2) analyses on all bi-monthly serial MRI time points of the study.

The LVs are in milliliters.

**Table 4:** Relationship between cortical volume and disability progression over 2 years in patients with relapsing-remitting multiple sclerosis.

Disability progression	Coefficient	Standard Error	*T*	*P* value
Disease duration	0.04	0.015	2.99	0.003
CV	−0.001	0.0004	−2.57	0.01

Disease duration	0.05	0.015	3.11	0.002
WBV	−0.0003	0.0002	−1.55	0.12

Disease duration	0.05	0.015	3.11	0.002
T2-LV	0.005	0.004	1.21	0.23

CV: cortical volume, WBV: whole brain volume, and LV: lesion volume.

The statistical analysis between CV and disability progression, as measured by EDSS, was performed using mixed effect model analyses on all bi-monthly serial MRI time points of the study over 2 years.

When adjusted for baseline CV, by using the changes in CV from baseline and every six months in our analysis, the association with disability progression became nonsignificant in both early and late RRMS groups (*P* = 0.13).

The LVs are in milliliters.
